# Impact of cigarette smoking on fluorescence lifetime of ocular fundus

**DOI:** 10.1038/s41598-023-37484-4

**Published:** 2023-07-17

**Authors:** Svenja Rebecca Sonntag, Marie Kreikenbohm, Giulia Böhmerle, Jessica Stagge, Salvatore Grisanti, Yoko Miura

**Affiliations:** 1 Department of Ophthalmology, University of Lübeck, Ratzeburger Allee 160, 23538 Lübeck, Germany; 2 Institute of Biomedical Optics, University of Lübeck, Lübeck, Germany; 3 Medical Laser Center Lübeck, Lübeck, Germany

**Keywords:** Diagnostic markers, Translational research

## Abstract

Cigarette smoking is known to adversely affect cellular metabolism and is a risk factor for various retinal diseases. Fluorescence lifetime imaging ophthalmoscopy (FLIO) has the potential to detect metabolic changes in the ocular fundus. Aim of this study was to analyze the influence of cigarette smoking on fluorescence lifetime (FLT) of healthy eyes using FLIO. Twenty-six non-smokers and 28 smokers aged between 20 and 37 years without systemic and ocular diseases were investigated by FLIO (excitation: 473 nm, emission: short spectral channel (SSC) 498–560 nm, long spectral channel (LSC) 560–720 nm). The FLT at the ETDRS grid regions were analyzed and compared. In SSC, the mean FLT (τ_m_) of smokers was significantly longer in the ETDRS inner ring region, whereas the τ_m_ in LSC was significantly shorter in the outer ring. For the long component (τ_2_), smokers with pack year < 7.11 showed significantly shorter τ_2_ in SSC than non-smokers and the smokers with pack year ≥ 7.11. There were no significant differences in retinal thickness. The lack of obvious structural differences implies that the observed FLT changes are likely related to smoking-induced metabolic changes. These results suggest that FLIO may be useful in assessing retinal conditions related to lifestyle and systemic metabolic status.

## Introduction

Cigarette smoking is one of the leading preventable causes of morbidity and mortality worldwide^1^. According to the Global Tobacco Surveillance System (GTSS), which was founded in 1998 by the World Health Organization (WHO), the Centers for Disease Control and Prevention (CDC), and the Canadian Public Health Association (CPHA) for the monitoring of the worldwide smoking behaviors^1^ the global prevalence of cigarette use is 22.3% (data of 2020, 36.7% for males, 7.8% for females)^2^. Although WHO reports a declining global smoking prevalence, the goal of a 30% reduction by 2025 relative to 2010 won’t be reached^2^.

Smoking has been proven by many studies to negatively affect cellular function and metabolism throughout the body^3,4^. Therefore, has a great impact not only on the health of lungs, but also of the ocular tissues including the retina^5^. Earlier studies revealed that cigarette smoking increases the risk of ocular diseases, such as age-related macular degeneration (AMD), glaucoma, uveitis or Grave’s ophthalmopathy^5–7^. Although there are studies on the underlying mechanisms of retinal pathologies induced by cigarette smoking^8–10^, its influence on retinal metabolism and early disease development in young healthy eyes is still not fully understood. An earlier study with OCT-angiography showed a reduced deep retinal vascular density in healthy eyes of smokers^11^. However, with respect to cellular metabolism, there is currently no method to assess the effects of smoking on fundus tissue.

Fluorescence lifetime imaging ophthalmoscopy (FLIO) is a new diagnostic tool that measures the fluorescence lifetime (FLT) of retinal intrinsic fluorophores non-invasively^12–14^. FLIO utilizes a 473 nm laser to excite fluorophores, detects and counts emitted fluorescence at wavelength from 498 to 720 nm. FLT is defined as the time until the fluorescence intensity drops down to 1/e (about 37%) of the initial value, usually in the range of picosecond (ps) to nanosecond (ns). The FLT is fluorophore-specific and independent of their fluorescence intensity. Natural fluorophores in the fundus tissue that could be excited at FLIO include bisretinoids, collagen, elastin, some coenzymes, macular pigments and melanin^12,13,15,16^. Among them, the coenzymes essential to cell energy metabolism, such as flavin adenine dinucleotide (FAD) and flavin mononucleotide (FMN) are known to change their FLT depending on its protein-binding states, that may alter with change in the state of cell energy metabolic state^17^.

The FLT can thus be altered not only by changes in the composition of fluorophores due to structural changes in fundus tissue, but also by cell metabolic changes. Our previous ex vivo study showed the change in FLT of RPE under sublethal oxidative stress or after laser irradiation^16,18^. An in vivo study rather showed transient changes in FLT around selectively treated laser spots^19^. As for clinical study, patients with type 2 diabetes without diabetic retinopathy^20^ as well as patients with Alzheimer’s disease^21^ revealed changes in FLT without structural changes, which underlines the potential of FLIO in the observation of metabolic disease mechanisms.

Therefore, aim of this study was to conduct the FLIO of healthy eyes of subjects with and without a habit of cigarette smoking, to see if it may imply potential metabolic alterations induced by smoking behavior.

## Methods

### Study design

This cross-sectional study was conducted at the Department of Ophthalmology of the University Medical Center Schleswig–Holstein, Campus Lübeck, Germany. A total number of 54 participants, 26 non-smokers and 28 smokers, were enrolled between April 2021 and September 2021. The study was positively accepted by the ethics committee of the University of Lübeck and conducted in accordance with the ethical standards stated in the Declaration of Helsinki. All subjects were informed about the study in written and oral form before written informed consent was obtained from them.

### Participants

Both male and female healthy subjects were included in the study regardless of ethnicity and refractive error. Inclusion criteria for study participation were the ability to consent, healthy retinal findings, and subject age between 20 and 40 years. Exclusion criteria were subjects with retinal diseases, relevant media opacity, condition after eye surgery, and a narrow chamber angle that would not allow the drug-induced mydriasis. No systemic diseases such as thyroid dysfunction or other hormonal disorders, diabetes mellitus or hypertension were allowed to be present. Due to altered hormone levels, pregnant or lactating women were also excluded from the study. Furthermore, subjects suffering from epilepsy or subjects who were unable to cooperatively participate in the examinations were not allowed to be included in the study. Based on a previous literature^22^, the group of smokers was defined as those who smoked five cigarettes daily for at least two years.

### Procedure

After informed consent a subject questionnaire to collect personal data was completed. The status of various previous diseases, such as arterial hypertension, diabetes mellitus, depression, hormonal disorders, hyperlipidemia, sleep disorders, and disorders affecting the eye and retina, was queried. A medication history, lifestyle history, and nicotine history were performed in detail.

After completion of the written formalities and the questionnaire, the slit lamp microscopy was performed including confirmation of the anterior chamber angle. Measurement of intraocular pressure by non-contact air tonometry and refractometry, a measurement of the best correlated visual acuity (BCVA) were subsequently performed. If the findings were unremarkable, the pupils were dilated bilaterally with eye drop of tropicamide and phenylephrine.

In medicated mydriasis, fundus ophthalmoscopy, optical coherence tomography (OCT; Spectralis OCT, Heidelberg Engineering, Heidelberg, Germany) of macula were performed. Consequently, FLIO was performed as described later in detail. In case of detection of abnormalities in fundus, affected subjects would not have been included in the evaluation. However, this did not occur because no pathologic findings were seen in any subject. The retinal thickness was obtained from the data of macular OCT. The early treatment diabetic retinopathy study (ETDRS) grid for analysis was defined in the same way as for the FLIO. It was centered over the fovea and divided into a central area (C), the nasal area (N), the temporal area (T), the superior area (S) and the inferior area (I) with an inner and an outer part. The three rings had a diameter of 1 mm, 3 mm and 6 mm. The average retinal thickness of each area, as calculated by the software, was used for the analysis.

### Fluorescence lifetime imaging ophthalmoscopy (FLIO)

The FLT of the fundus autofluorescence was measured with the FLIO, using a system provided by Heidelberg Engineering (Heidelberg, Germany). FLIO utilizes a picosecond (70 ps)-pulsed excitation laser (473 nm) with a repetition rate of 80 MHz to excite fluorophores, and the FLT measurement is based on the to time-correlated single photon counting (TCSPC) technique. Two highly sensitive hybrid detectors (HPM-100-40; Becker & Hickl, Berlin, Germany) register the detected emission photons in a short spectral channel (SSC: 498–560 nm) and a long spectral channel (LSC: 560–720 nm) that are connected to the TCSPC module (SPC-150, Becker & Hickl) for photon counting. Infrared laser (815 nm) eye tracking system compensates for eye movements during measurement. Image acquisition took place in a darkened room until 1000 photons per pixel were detected at the fovea centralis in both channels.

### FLIO data analysis

For analysis of the detected photon counts the data was processed in the software SPCImage (version 8.0 NG, Becker & Hickl). A detailed description of this software can be found elsewhere^23^. The measured TCSPC decay was fitted to a biexponential curve with a binning factor of 1. This curve can be described by the sum of its exponential components:

$$f\left(t\right)=\sum_{n=1}^{2}{\alpha }_{i}\cdot {e}^{-t/{\tau }_{i}}$$

where ${\tau }_{1}$ and ${\tau }_{2}$ describes the FLTs of short and long exponential components, respectively, and ${\alpha }_{1}$ and ${\alpha }_{2}$ are the corresponding amplitudes. Furthermore, the software SPCImage calculates the mean FLT ${\tau }_{m}$ of a biexponential fitting model as followed:

$${\tau }_{m}=\frac{{\alpha }_{1}\cdot {\tau }_{1}+{\alpha }_{2}\cdot {\tau }_{2}}{{\alpha }_{1}+{\alpha }_{2}}$$

**Figure 1:**
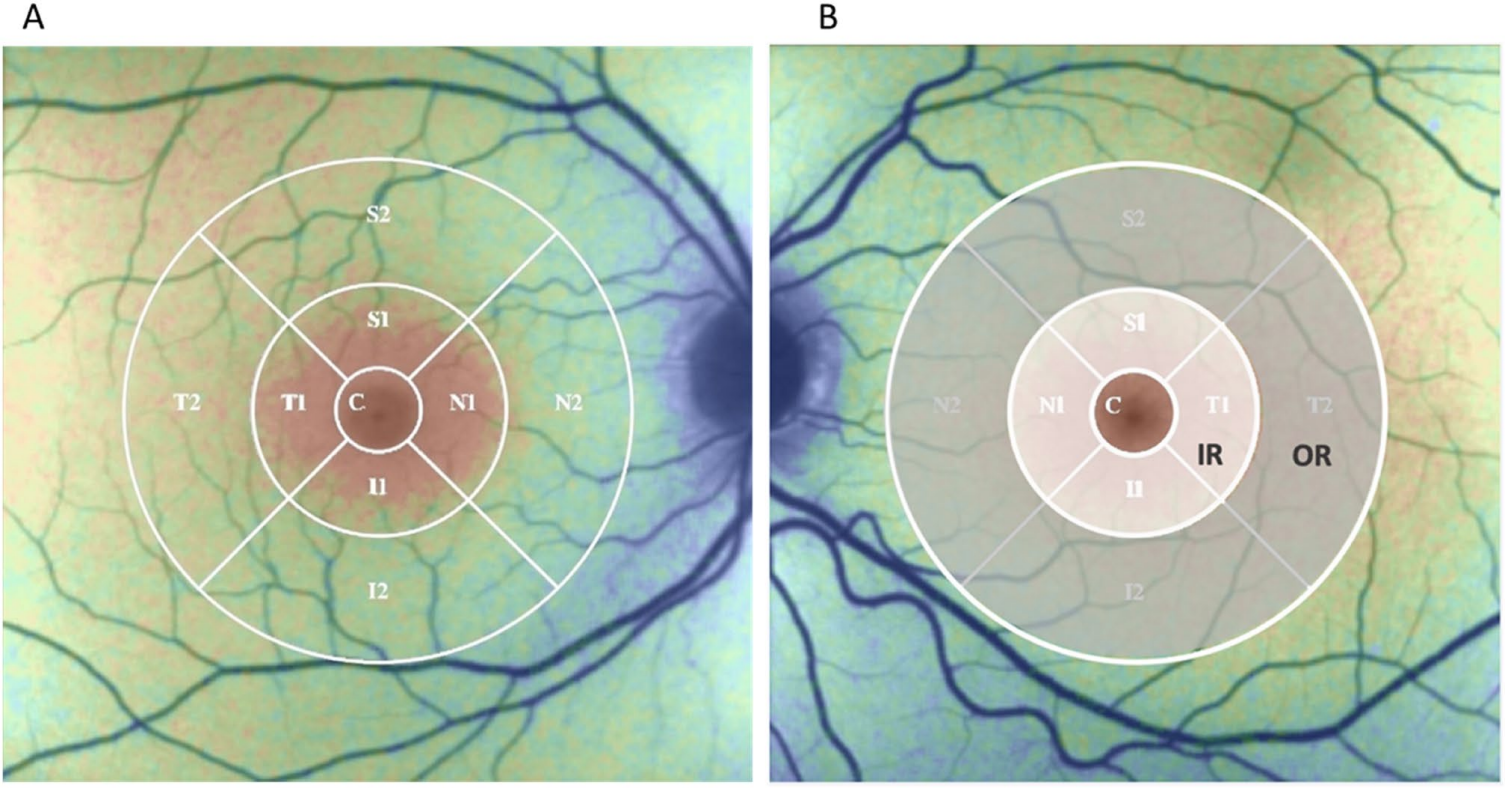
ETDRS grid in the FLIO-reader software showing different regions for analysis. (**A**) 9 small subareas; C: central fovea, N: nasal, I: inferior, T: temporal, S: superior. 1 and 2 indicate inner and outer areas, respectively. (**B**) 3 large subareas; C: central fovea, IR: inner ring, OR: outer ring.

The obtained values of the FLT parameters (τ_1_, τ_2_, τ_m_) for each pixel position and the intensity image from both channels from both eyes were exported for further analysis in the software FLIO-reader (ARTORG Center for Biomedical Engineering Research, University of Bern, Switzerland). An integrated standardized ETDRS grid, with rings of Central Area (C), Inner Ring (IR) and Outer Ring (OR), with diameters approximately 1 mm, 3 mm, and 6 mm, respectively, may facilitate calculation of the mean and standard deviation of the FLT parameters for each area. For this study the ETDRS grid was centered over the fovea. Furthermore, the IR and OR were divided into 4 sub-areas, nasal (N), superior (S), temporal (T) and inferior (I) (Fig. 1).

### Statistical analysis

The statistical analysis was performed with the statistical program Jamovi (version 2.3.0). For the metric data, the mean, median, and standard deviations were calculated. Normality of distribution was tested with Q–Q plots, and comparisons between two groups was conducted with a t-test for unpaired samples. Comparisons among three groups was conducted with a repeated measures analysis of variance (ANOVA) followed by the post hoc test for parametric data, and Kruskal–Wallis test was applied for non-parametric data. For the gender data, chi-square test for independence was used.

For the analysis of FLT parameters, ANOVA followed by the post hoc test was performed. Correlation analyses were performed for interval-scaled variables according to Pearson (r). The interpretation of the correlation coefficient was according to Cohen. Pearson’s correlation was also applied for the correlation of the right and left eyes for each FLT parameters (τ_1_, τ_2_, τ_m_) for both channels. Values between 0.1 ≤ r < 0.3 were defined as small, 0.3 ≤ r < 0.5 as medium, and of r ≥ 0.5 as large. A *p*-value of < 0.05 was set as the significance threshold.

During the analysis, the smoker group was further divided into two groups based on the „pack-year”, defined as (packs smoked per day) × (years as a smoker). This value is widely used as a measure of cumulative cigarette intake^24^. For this purpose, the obtained median value of the pack-year among the smokers was used as a basis for dividing the group.

The statistical analysis and the applied tests were reviewed in collaboration with the Institute of Medical Biometry and Statistics (IMBS) at the University of Lübeck.

## Results

### General data

As shown in Table 1, the mean age ± standard deviation (SD) of non-smokers was 26.7 (± 4.1) years, whereas the smokers were 28.5 (± 4.7) years old, which showed no significant difference (*p > *0.05). Gender distribution in the non-smoker group was homogeneous with 50% females and 50% males. In the smoker group, there are slightly more male subjects with 53.6% males and 46.4% females. Comparing the BMI of both groups, the mean BMI in the smoker group of 26.6 (± 5.4) kg/m^2^ was significantly higher than that of the non-smoker group of 22.8 (± 2.6) kg/m^2^ (p = 0.002). The smoker group has smoked for a mean of 12.0 (± 4.9) years and when calculated in pack-year, the mean value was 8.0 (± 6.1) (median: 7.11, inter-quartile range (IQR) 8.1), while these numbers for the non-smoker group was zero. Based on the median value of pack-year, the smoker group was further divided into two subgroups to examine smoking dose–response relationships; Group 1 composed of the smokers with pack-year less than 7.11 and Group 2 includes the smokers with pack-year of 7.11 or more. This divided the group of 28 smokers into two groups of n = 14. The non-smoker group is referred to here as Group 0. When divided in this way, the age of Group 2 was significantly higher than Group 0 and Group 1 (Mean age: 26.7, 26.6, and 30.5 for Group 0, 1 and 2, respectively). BMI of both smoking subgroups was also significantly larger than in the non-smoker group, as in the two-group analysis. (Table 2).

### Ophthalmologic data

The mean of the intraocular pressure (IOP) of non-smokers was 14.6 (± 3.3) and 14.5 (± 3.2) mmHg in right and left eye, respectively. In comparison, the values of the smokers were 16.3 (± 2.8) and 16.1 (± 2.5) mmHg in right and left eye, respectively, that were significantly higher than the values of the non-smoker group (*p <* 0.05). There was no difference in the IOD values between both eyes. The refraction of both groups was generally not significantly different. (Table 1). Retinal thickness (macular thickness: MT) measured by OCT did not show significant differences between both groups in any eye (supplementary Table S1).

**Table 1:** Summary of general as well as ophthalmologic data of the subject population comparing between the non-smokers’ and smokers’ groups and statistical results (p values) for group comparison; SD: standard deviation, IQR: interquartile range, OD: oculus dexter (right eye), OS: oculus sinister (left eye), IOP: intraocular pressure, Sph: sphere, Cyl: cylinder, MT: macular thickness, C, N1, N2, S1, S2, T1, T2, I1, I2, IR, OR: as explained in Fig. 1. *p < 0.05, **p < 0.01, ***p < 0.001.

Parameter	Unit	Non-Smokers (n = 26)			Smokers (n = 28)			*p*
		Mean (SD)	Median	IQR	Mean (SD)	Median	IQR	
Age	(Years)	26.7 (4.1)	26.5	23.0 to 30.3	28.5 (4.7)	28.0	25.0 to 32.0	0.130
Male	(No. of subject)	13			15			0.793
Female	(No. of subject)	13			13			
BMI	(kg/m^2^)	22.8 (2.6)	23.1	20.3 to 24.3	26.6 (5.4)	25.0	22.7 to 30.1	0.002**
Years-smoked	(years)	0	0	0	12.0 (4.9)	10.8	9.0 to 15.8	***
pack-year		0	0	0	8.0 (6.1)	7.11	2.4 to 11.7	***
IOP OD	(mmHg)	14.6 (3.3)	15.0	12.5 17	16.3 (2.3)	16.0	15.0 to 18.0	0.038*
IOP OS	(mmHg)	14.5 (3.2)	15.0	11.8 to 17	16.1 (2.5)	16.0	14.0 to 18.0	0.041*
Sph OD	(dpt)	− 1.1 (1.8)	− 0.75	− 2.1 to 0	− 0.67 (2.0)	− 0.63	− 1.6 to 0.5	0.380
Sph OS	(dpt)	− 1.1 (1.9)	− 0.63	− 1.8 to 0.25	− 0.63 (1.8)	0.13	− 1.4 to 0.5	0.326
Cyl OD	(dpt)	− 0.5 (0.4)	− 0.38	− 0.56 to − 0.25	− 0.69 (0.58)	− 0.5	− 1 to − 0.25	0.171
Cyl OS	(dpt)	− 0.5 (0.3)	− 0.5	− 0.75 to − 0.25	− 0.83 (0.73)	− 0.5	− 1 to − 0.25	0.043*

**Table 2:** Summary of general as well as ophthalmologic data of the subject population of all groups after dividing the smokers group in two groups by the median value of the pack-year (PY) 7.1, and statistical results (p values) for group comparison. Group 0: non-smokers, Group 1: smokers with PY < 7.1. Group 2: smokers with PY ≥ 7.1. SD: standard deviation, IQR: interquartile range, OD: oculus dexter (right eye), OS: oculus sinister (left eye), IOP: intraocular pressure, Sph: sphere, Cyl: cylinder, MT: macular thickness, C, N1, N2, S1, S2, T1, T2, I1, I2, IR, OR: as explained in Fig. 1. *p < 0.05, **p < 0.01, ***p < 0.001.

Parameter	Unit	Group 0 (G0) Non-smokers (n = 26)	Median	IQR	Group 1 (G1) Smokers, PY < 7.11 (n = 14)	Median	IQR	Group 2 (G2) Smokers, PY ≥ 7.11 (n = 14)	Median	IQR	p (Anova/Chi^2^)	p (Post hoc)	G0 vs G2	G1 vs G2
		Mean (SD)			Mean (SD)			Mean (SD)				G0 vs G1		
Age	(Years)	26.7 (4.1)	26.5	23.0 to 30.3	26.6 (3.5)	26	24 to 30	30.5 (5.1)	31.5	25 to 35.3	0.02*	0.93	0.01**	0.02*
Male	No. of subject	13			7			8			0.90			
Female	No. of subject	13			7			6						
BMI	(kg/m^2^)	22.8 (2.6)	23.1	20.3 to 24.3	27.8 (5.0)	27.4	22.7 to 29.2	26.4 (5.9)	23.7	22.7 to 31.8	0.03*	0.01**	0.02*	0.81
Years-smoked	(years)	0	0	0	9.2 (3.4)	9.0	5.8 to 10.9	14.7 (4.7)	13.8	10.8 to 19.3	***	***	***	***
pack-year		0	0	0	3.2 (1.6)	2.4	2.1 to 4.6	12.8 (5.0)	11.2	9.0 to 16.87	***	***	***	***
IOP OD	(mmHg)	14.6 (3.3)	15.0	12.5 to 17	17.3 (2.4)	18	15 to 19.3	15.3 (1.7)	15.5	14.0 to 16.0	0.03*	0.01**	0.47	0.06
IOP OS	(mmHg)	14.5 (3.2)	15.0	11.8 to 17	17.2 (2.3)	16.5	16.0 to 19.3	15.0 (2.2)	14.0	13.8 to 17.3	0.01**	0.004**	0.56	0.04*
Sph OD	(dpt)	− 1.1 (1.8)	− 0.75	− 2.1 to 0	− 0.63 (2.36)	− 0.63	− 1.81 to 0.56	− 0.71 (1.68)	− 0.38	− 1.56 to 0.31	0.68	0.43	0.52	0.90
Sph OS	(dpt)	− 1.1 (1.9)	− 0.63	− 1.8 to 0.25	− 0.88 (2.13)	− 0.88	− 1.94 to 0.56	-0.38 (1.43)	0.25	− 0.81 to 0.31	0.48	0.69	0.23	0.48
Cyl OD	(dpt)	− 0.5 (0.41)	− 0.375	− 0.56 to − 0.25	− 0.64 (0.55)	− 0.50	− 1 to − 0.25	− 0.73 (0.61)	− 0.50	− 1.25 to − 0.25	0.36	0.40	0.17	0.64
Cyl OS	(dpt)	− 0.51 (0.33)	-0.50	− 0.75 to − 0.25	− 0.73 (0.60)	− 0.50	− 1 to − 0.25	− 0.93 (0.87)	− 0.63	− 1.40 to − 0.25	0.32	0.25	0.03*	0.37

### FLIO

**Figure 2:**
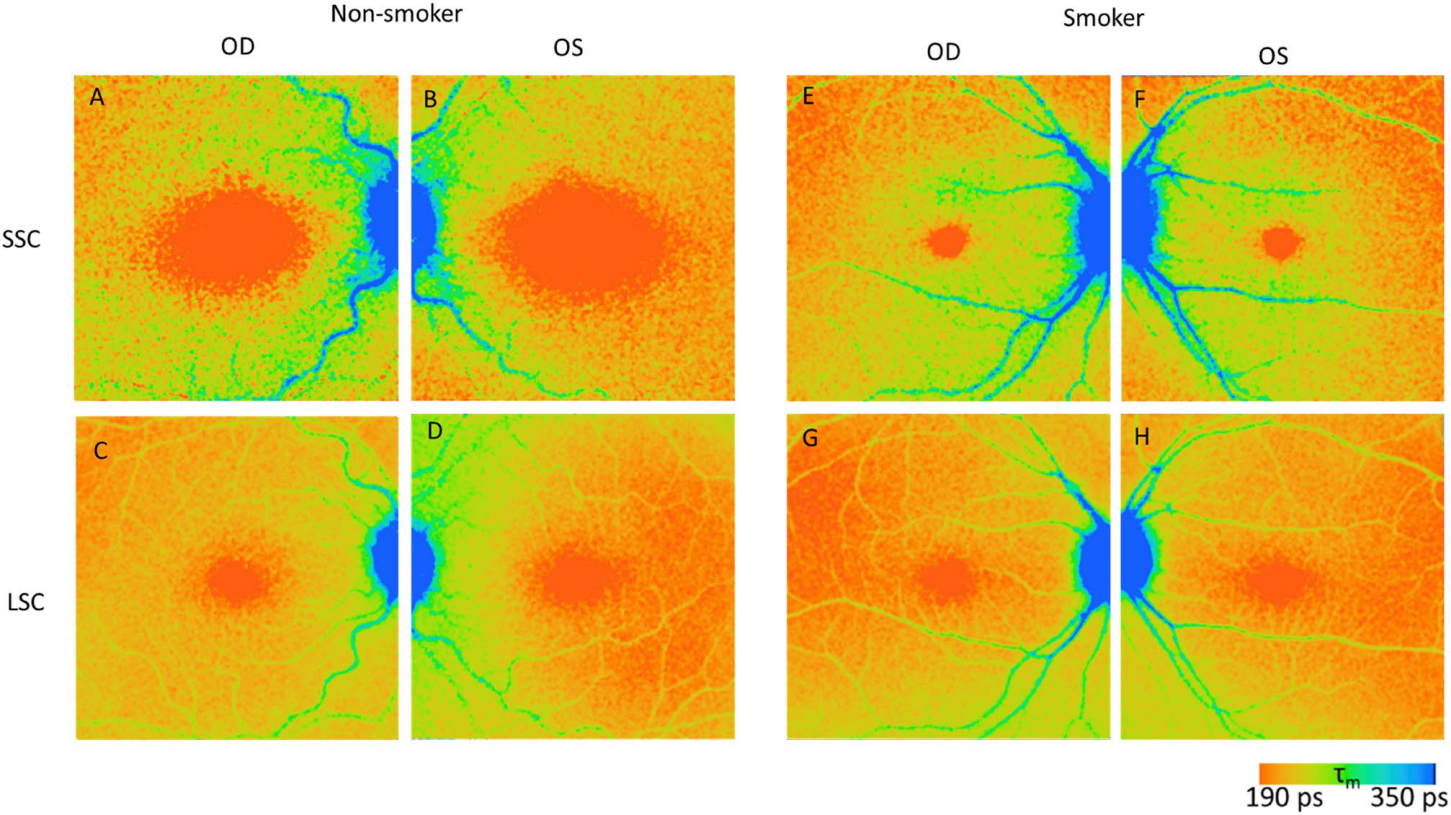
Representative FLIO images of a non-smoker (**A**–**D**) and a smoker (**E**–**H**), demonstrating the value of mean FLT (τ_m_) in pseudocolor. SSC (short spectral channel) (**A**, **B**, **E**, **F**), LSC (long spectral channel) (**C**, **D**, **G**, **H**).

Figure 2 shows a representative FLIO image (mean FLT: τ_m_) of a non-smoker and a smoker. FLIO allows the values of the examined parameters to be displayed in a pseudo-color image. Typically, small values are displayed in orange and large values in blue. The foveal area shows a shorter τ_m_ than other areas due to the macular pigment^25^, and the optic nerve and blood vessels show a significantly longer τ_m_ than other retinal areas.

For data analysis, the program FLIO-reader was used to evaluate the mean values for each region of the ETDRS grid, as shown in Fig. 1. In the following, measurement results of τ_m_ and each FLT component (τ_1_, τ_2_) for each spectral channel (SSC, LSC) are presented. Previous study results revealed that there is no difference in τ_m_ between right and left eyes^26^. In the present study, FLTs of both eyes were also compared by channel and by component, and no significant differences were found. Furthermore, there was a high correlation between right and left eyes regarding each FLT parameter (τ_1_, τ_2_, τ_m_) for three main ETDRS areas (C, IR and OR) in both channels (18 items in total), where the pearson coefficient r was > 0.8 for all items (*p <* 0.0001) (Supplementary Table S2, Supplementary Fig. 1) Therefore, for the sake of clarity, we present here a series of results only for the right eye.

**Figure 3:**
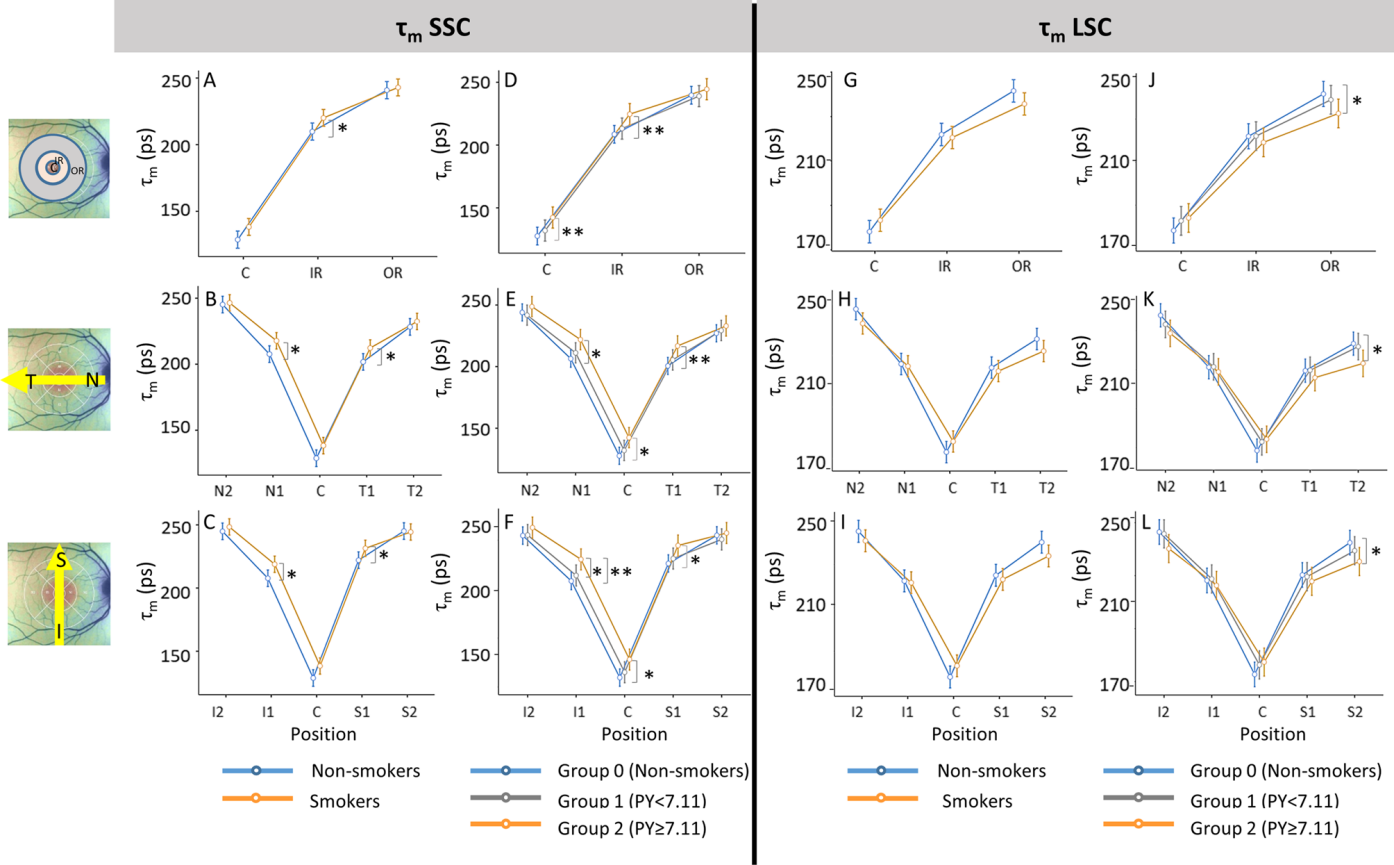
Mean with confidence interval for the mean fluorescence lifetime τ_m_ in the short spectral channel (SSC) and long spectral channel (LSC) of the right eye. The top row (**A**, D, **G**, **J**) show the values for the central (C), inner ring (IR), and outer ring (OR); the middle row (**B**, **E**, **H**, **K**) show the values for areas on the horizontal line (N2, N1, C, T1, T2); and the bottom row (**C**, **F**, **I**, **L**) show the values for areas on the vertical line (I2, I1, C, S1, S2). The graphs on the left for each channel (**A**–**C**, **G**–**I**) compare non-smokers and smokers, and on the right (**D**–**F**, **J**–**L**) compare non-smokers (Group 0) and smokers divided in two groups (Group 1: pack-year < 7.11, Group 2: pack-year ≥ 7.11). *p < 0.05. The numerical data for the basis of the graphs and statistic results of repeated measures ANOVA and post hoc tests are shown in Table 3.

**Figure 4:**
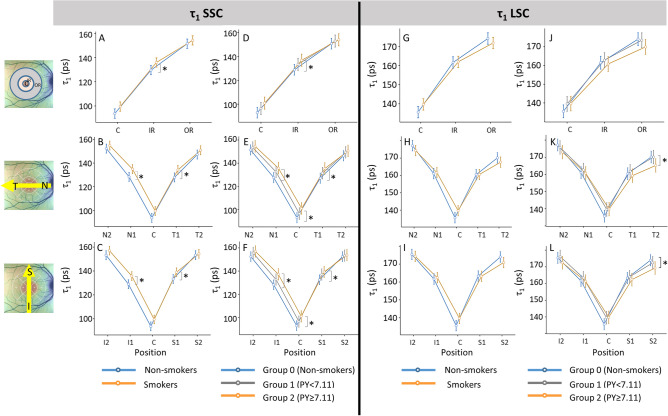
Mean with confidence interval for the shorter component τ_1_ in the short spectral channel (SSC) and long spectral channel (LSC) of the right eye. The top row (A, D, G, J) show the values for the central (C), inner ring (IR), and outer ring (OR); the middle row (B, E, H, K) show the values for areas on the horizontal line (N2, N1, C, T1, T2); and the bottom row (**C**, **F**, **I**, **L**) show the values for areas on the vertical line (I2, I1, C, S1, S2). The graphs on the left for each channel (**A**–**C**, **G**–**I**) compare non-smokers and smokers, and on the right (**D**–**F**, **J**–**L**) compare non-smokers (Group 0) and smokers divided in two groups (Group 1: pack-year < 7.11, Group 2: pack-year ≥ 7.11). *p < 0.05, **p < 0.01. The numerical data for the basis of the graphs and statistic results of repeated measures ANOVA and post hoc tests are shown in supplementary Table S3.

**Figure 5:**
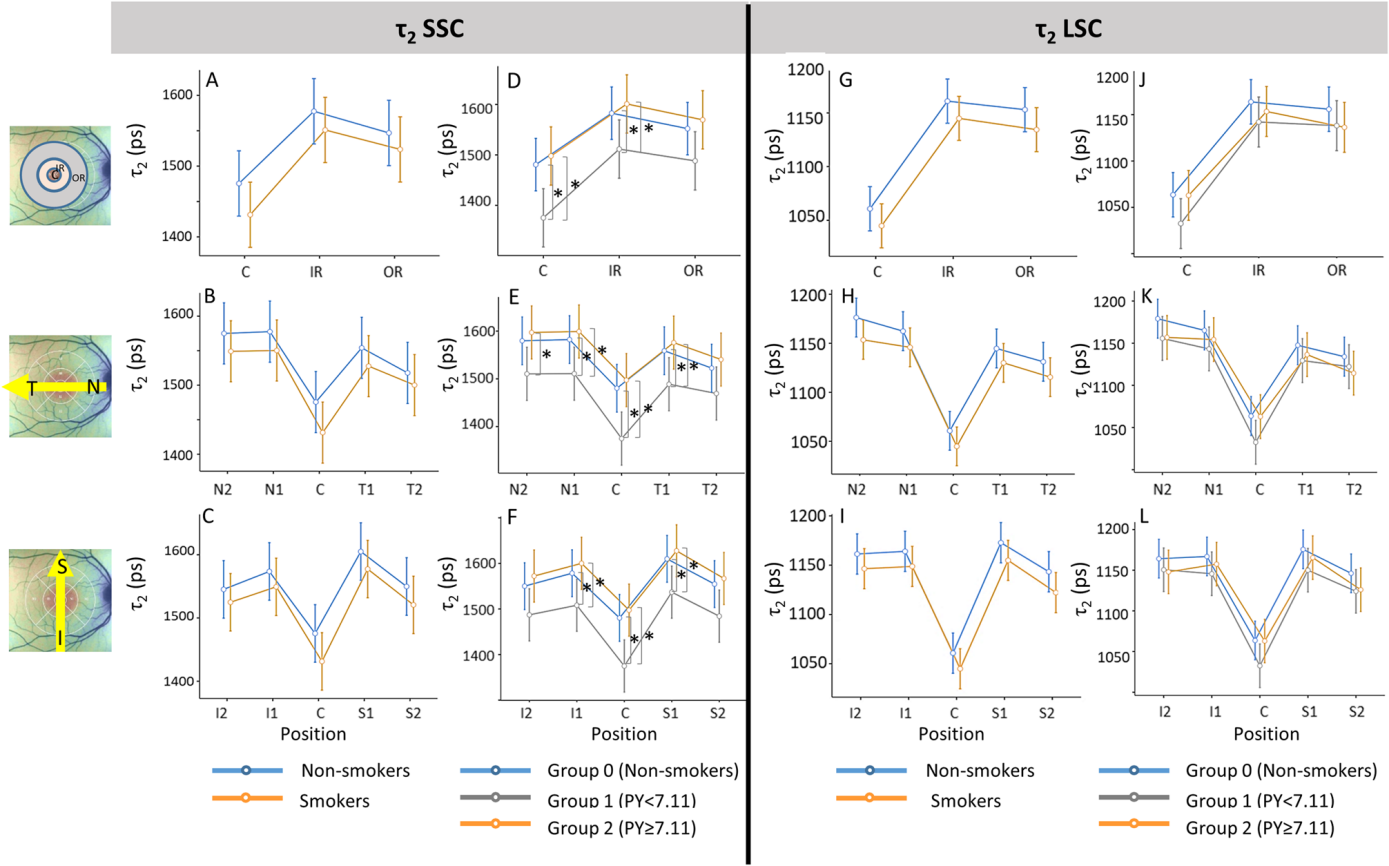
Mean with confidence interval for the component τ_2_ in the short spectral channel (SSC) and long spectral channel (LSC) of the right eye. The top row (**A**, **D**, **G**, **J**) show the values for the central (**C**), inner ring (IR), and outer ring (OR); the middle row (**B**, **E**, **H**, **K**) show the values for areas on the horizontal line (N2, N1, C, T1, T2); and the bottom row (**C**, **F**, **I**, **L**) show the values for areas on the vertical line (I2, I1, C, S1, S2). The graphs on the left for each channel (**A**–**C**, **G**–**I**) compare nonsmokers and smokers, and on the right (**D**–**F**, **J**–**L**) compare non-smokers (Group 0) and smokers divided in two groups (Group 1: pack-year < 7.11, Group 2: pack-year ≥ 7.11). *p < 0.05, **p < 0.01. The numerical data for the basis of the graphs and statistic results of repeated measures ANOVA and post hoc tests are shown in supplementary Table S4.

In the followings, the results for τ_m_ and its components τ_1_ and τ_2_ in SSC and LSC are separately described. Graphs in Figs. 3, 4, and 5 show the mean values and confidence intervals of τ_m_, τ_1_ and τ_2_, respectively, at specific regions in three different views; A, D, G and J of the three figures show the values in the foveal region (C), inner ring (IR), and outer ring (OR), the B, E, H and K are of the horizontal section, i.e., from nasal (N2) to temporal (T2), and C, F, I and L are of the vertical section, i.e., from inferior (I2) to superior (S2) regions.

**Table 3:** Mean ± standard deviation of τ_m_ (in ps) in SSC (top) and in LSC (bottom) for each area of ETDRS grid in the right eye of the groups of non-smokers and smokers, p-values from post hoc test after repeated measure ANOVA, and the results of correlation analysis (r: correlation coefficient) between the τ_1_ in SSC and the number of pack-year, years-smoked and retinal thickness at the corresponding area. * *p <* 0.05, ** *p <* 0.01, *** *p <* 0.001.

	Area	τ_m_ (ps)		Post hoc p	τ_m_ (ps)			Post hoc p			Correlation coefficient (r)			Area
		Non-smokers (n = 26)	Smokers (n = 28)		Group 0 (G0) Non-smokers (n = 26)	Group 1(G1) Smokers PY < 7.11 (n = 14)	Group 2 (G2) Smokers PY ≥ 7.11 (n = 14)	G0 vs G1	G0 vs G2	G1 vs G2	Pack-year	Years -smoked	Retinal thickness	
SSC	C	129 ± 17.7	138 ± 20.1	0.071	129 ± 17.7	133 ± 16.8	143 ± 22.4	0.488	0.022*	0.150	0.424**	0.342*	0.099	C
	N1	207 ± 19.4	218 ± 16.7	0.044*	207 ± 19.4	212 ± 14.8	223 ± 17.2	0.425	0.011*	0.115	0.347*	0.367**	0.074	N1
	N2	245 ± 14.3	246 ± 14.9	0.75	245 ± 14.3	243 ± 11.7	250 ± 17.3	0.651	0.330	0.213	0.140	0.194	0.195	N2
	S1	222 ± 19.4	231 ± 14.1	0.046*	222 ± 19.4	226 ± 11.5	237 ± 14.8	0.465	0.010*	0.094	0.360**	0.332*	0.025	S1
	S2	245 ± 17.9	244 ± 15.8	0.874	245 ± 17.9	242 ± 14.3	247 ± 17.2	0.553	0.738	0.417	0.042	0.028	0.276*	S2
	T1	202 ± 16.1	212 ± 15.5	0.018*	202 ± 16.1	206 ± 14.0	218 ± 15.3	0.343	0.003**	0.057	0.436***	0.438***	0.019	T1
	T2	228 ± 14.3	232 ± 12.8	0.270	228 ± 14.3	230 ± 11.7	234 ± 13.9	0.616	0.193	0.478	0.181	0.184	0.331*	T2
	I1	208 ± 17.7	219 ± 16.6	0.021*	208 ± 17.7	212 ± 13.7	225 ± 17.1	0.412	0.002**	0.041*	0.428**	0.442***	0.028	I1
	I2	245 ± 14.7	248 ± 15.4	0.400	245 ± 14.7	245 ± 14.0	251 ± 16.6	0.932	0.196	0.288	0.195	0.224	0.350*	I2
	Inner Ring	210 ± 17.7	220 ± 15.2	0.026*	210 ± 17.7	214 ± 12.7	226 ± 15.8	0.396	0.004**	0.064	0.403**	0.405**	0.036	Inner Ring
	Outer Ring	241 ± 14.4	243 ± 13.9	0.600	241 ± 14.4	240 ± 12.0	246 ± 15.5	0.881	0.314	0.311	0.144	0.162	0.295*	Outer Ring

	Area	τ_m_ (ps)		Post hoc p	τ_m_ (ps)			Post hoc p			Correlation coefficient (r)			Area
		Non-smokers (n=26)	Smokers (n=28)		Group 0 (G0) Non-smokers (n=26)	Group 1(G1) Smokers PY <7.11 (n=14)	Group 2 (G2) Smokers PY ≥7.11 (n=14)	G0 vs G1	G0 vs G2	G1 vs G2	Pack-year	Years -smoked	Retinal thickness	
LSC	C	178 ± 12.8	183 ± 14.7	0.177	178 ± 12.8	183 ± 16.6	184 ± 13.1	0.336	0.215	0.803	0.288*	0.291*	0.083	C
	N1	221 ± 10.7	220 ± 14.0	0.753	221 ± 10.7	221 ± 16.3	219 ± 11.8	0.959	0.573	0.590	0.054	0.115	0.039	N1
	N2	248 ± 11.7	241 ± 17.5	0.095	248 ± 11.7	243 ± 18.8	238 ± 16.6	0.357	0.069	0.421	− 0.101	− 0.025	0.052	N2
	S1	225 ± 10.4	223 ± 12.5	0.550	225 ± 10.4	224 ± 14.3	222 ± 10.8	0.839	0.440	0.617	0.037	0.056	0.011	S1
	S2	240 ± 11.1	234 ± 13.7	0.068	240 ± 11.1	237 ± 15.1	231 ± 12.2	0.364	0.036*	0.282	− 0.121	− 0.101	0.039	S2
	T1	219 ± 10.6	217 ± 13.2	0.610	219 ± 10.6	219 ± 15.8	216 ± 10.4	0.961	0.378	0.414	0.028	0.052	− 0.071	T1
	T2	233 ± 11.8	227 ± 15.1	0.121	233 ± 11.8	232 ± 17.9	223 ± 10.6	0.730	0.026*	0.095	− 0.140	− 0.128	0.019	T2
	I1	222 ± 10.1	222 ± 13.8	0.806	222 ± 10.1	223 ± 16.6	220 ± 10.8	0.861	0.565	0.510	0.064	0.126	− 0.033	I1
	I2	245 ± 12.2	241 ± 17.3	0.293	245 ± 12.2	244 ± 21.2	237 ± 12.2	0.861	0.121	0.225	− 0.047	0.033	0.118	I2
	Inner Ring	222 ± 10.2	221 ± 13.3	0.675	222 ± 10.2	222 ± 15.6	219 ± 10.8	0.982	0.480	0.523	0.047	0.090	− 0.016	Inner Ring
	Outer Ring	242 ± 11.2	236 ± 15.4	0.117	242 ± 11.2	239 ± 18.0	233 ± 12.1	0.547	0.048*	0.219	− 0.105	− 0.054	0.060	Outer Ring

Values of all parameters for non-smokers and smokers (total, subgroup), results of post hoc analyses, and correlation coefficient (r) between each FLT component and either pack-year, years-smoked, or retinal thickness are shown in the supplementary tables. For τ_m_, the numerical data for the basis of the graphs are shown in Table 3, and for τ_1_ and τ_2_ in the supplementary tables (Supplementary Tables S3 and S4).

#### τ_m_ (mean FLT)

In SSC, a significant difference in τ_m_ was detected between smokers and non-smokers in IR and its’ all subareas (N1, T1, I1, and S1), with the longer τ_m_ in the smoker group (Fig. 3A–C, Table 3, top). In the post hoc analysis with the divided smoking groups showed a significant difference in τ_m_ between Group 0 (non-smokers) and Group 2 (smokers with pack-year ≥ 7.11) at the fovea (C), in IR and each subarea of IR (Fig. 3D–F, Table 3, top)_._ A significant difference in τ_m_ between both smoker groups was also seen in I1 (Fig. 3F, Table 3, top). Correlation analysis showed a significant positive correlation between pack-year and τ_m_ as well as years-smoked and τ_m_ in the inner areas (C and areas in IR) (Table 3, top). A significant moderate positive correlation between retinal thickness and τ_m_ was found in S2, in T2, in I2, and the whole OR. However, τ_m_ of these areas showed no correlation with smoking behavior. (Table 3, top).

In LSC, no significant differences in τ_m_ were found between smokers and non-smokers in all regions (Fig. 3G–I). However, when smoker group was divided into two subgroups, significant differences in τ_m_ were found in OR, T2 and S2, between Group 0 and Group 2. Quite contrary to the results in SSC, τ_m_ of smokers with larger pack-year (Group 2) was significantly shorter than that of non-smokers. (Fig. 3J–L, Table 3, bottom). Correlation analysis showed a significant weak positive correlation between pack-year and τ_m_ as well as years_-_smoked and τ_m_ only in the fovea (C). There was no correlation between retinal thickness and τ_m_ in all regions. (Table 3, bottom).

#### τ_1_ (short FLT component)

In SSC, the smokers group showed significantly longer τ_1_ than the non-smokers in all areas of the IR (Fig. 4A–C, supplementary Table S3 top). Analysis with the two smoker groups showed a dose-dependent increase in the τ_1_, with significant differences in the fovea (C) and in the IR between Group 0 and Group 2 (Fig. 4D–F, supplementary Table S3, top).

Furthermore, there were moderate positive correlations in almost all regions between pack-year and τ_1_, and between the year-smoked and τ_1_ (Supplementary Table S3, top). Years-smoked tended to be slightly more strongly correlated with τ_1_ than pack-year. No significant correlation was found between retinal thickness and τ_1_ (supplementary Table S3, top).

In LSC, no significant difference in τ_1_ was found between non-smokers and smokers for all regions (Fig. 4G–I, supplementary Table S3, bottom). Analysis with the two smoker subgroups revealed a significant difference in τ_1_ in some subareas of the OR (T2 and S2), where τ_1_ of the smoker with higher pack-year was significantly shorter than the other two groups (Fig. 4J–L, supplementary Table S3, bottom).

Correlation analysis showed that pack-year and years-smoked showed a mild correlation with τ_1_ in the fovea (C), but not in the other regions. No correlation between retinal thickness and τ_1_ was found in all regions.

#### τ_2_ (long FLT component)

In SSC, when comparison was made between non-smokers and smokers, no significant difference in τ_2_ was detected in any area (Fig. 5A–C, supplementary Table S4, top). However, with dividing the smoker group into two subgroups, Group 1, the group of less pack-year, showed significantly shorter τ_2_ than other two groups in inner areas (C and all areas of IR) (Fig. 5D–F, supplementary Table S4, top). In the correlation analysis, there was no correlation between the pack-year or years-smoked with τ_2_ (supplementary Table S4, top). Some areas (T2, I1 and I2) showed a moderate positive correlation between retinal thickness and τ_2_, but not with smoking history.

In LSC, there were no significant differences in τ_2_ between non-smokers and smokers, and also among three groups with divided smoker groups in all areas (Fig. 5G–L, supplementary Table S4, bottom). There were also no significant correlations between pack-year, years-smoked or retinal thickness and τ_2_ in almost all regions (supplementary Table S4, bottom).

## Discussion

The retina is one of the most metabolically active tissues in the entire body, and at the same time, it is susceptible to light and other stresses. Therefore, its metabolic state is likely to be greatly affected by daily lifestyle. FLIO may facilitate understanding of the metabolic state of the retina, as the FLT of some intracellular fluorescent substances can be directly affected by their metabolic state. A previous study showed that the mean FLT (τ_m_) extends with increasing age, whereas pigmentation or gender did not show any correlation^27^. To date, however, no research has been done on the influence of lifestyle on FLIO.

It is a new insight that there was a statistically significant difference in the FLT between the non-smoker and smoker groups, and it indicates the potential of FLIO. Because the influence of endogenous parameters on FLIO is unknown except for age^27^, this study included young adults who were age-matched and had no history of systemic disease. Analysis of demographic data showed slight but statistically significant differences in BMI and IOP between the non-smoker and smoker groups. In addition, BMI was positively correlated with the amount of smoking (pack-year) (Pearson's r = 0.334). This is consistent with previous reports^28^. No study has yet pursued a pure correlation between BMI and retinal FLT. An evaluation in this regard would be also interesting, but it is not very meaningful to evaluate the correlation between BMI and FLT separately in this study, since the smoking factor cannot be excluded.

So far, observations on the effects of smoking on the retina have included structural changes such as retinal thickness with OCT^29^, vessel density with OCT angiography^11,30^, and changes in retinal blood flow using Doppler velocimetry^31^. However, no attempt has yet been made to explore metabolic changes in the retina.

In the present study, τ_1_ and τ_m_ showed a smoking dose-dependent elongation at the fovea and the inner ring in SSC, whereas, τ_2_ of light smokers (less pack-year) had a significantly shorter value in the fovea and inner ring at SSC than non-smokers and heavy smokers. There were no differences in the quality of the measurements and the fits, which could have led to this surprising result of τ_2_. Perhaps this result is related to mild metabolic or blood flow changes caused by light smoking. Indeed, there are reports on the protective effects of smoking^32,33^. We cannot speculate further at this time, but it is very interesting and warrants further investigation.

The link between cellular energy metabolism and FLT has been extensively studied in biological research^34,35^. The intracellular redox cofactors NADH and FAD are the most widely known, and are used as indicators of the state of energy metabolism, because their FLT can be significantly affected by their binding to enzymes^36,37^. In FLIO using 473 nm excitation light, NADH with its excitation spectrum in the UV region would be excluded, while FAD with its excitation peak around 456 nm could contribute to the results of FLT measurements in FLIO using blue light. A recent report by Kalinina et al. suggests that not only NADH and FAD, but also FLT of FMN should contribute significantly to FLT changes under cellular metabolic changes and should be considered in the interpretation^17^. Therefore, FLIO is not able to detect redox ratio, which requires measuring both NADH and FAD autofluorescence^34,35^, but it may be able to assess the metabolic state of cells by suggesting the state of flavins, which are known to contribute significantly to cellular metabolic activity and homeostasis^38,39^.

Other mechanisms, such as macular pigment density (MAPD) and vascular density, can also be considered relevant for the changes of FLT in SSC. Analysis of microstructural changes of habitual heavy smokers revealed a lower MAPD^40^. Since macular pigment such as lutein or zeaxanthin exhibit short FLT and predominantly measurable in the SSC^13,41^, a reduced MAPD could result in prolonged FLT in SSC. MAPD was not measured in this study, and the influence of MAPD levels on our results remains unknown, but since the significant difference in τ_m_ between non-smokers and somkers was observed in the inner ring and not in the central region, it seems unlikely that changes in macular pigment, which is most densely located in the central region, are significantly related to the present results.

As described above, smoking is also known to affect the blood flow in the retina and choroid^31,42^. Morgado *et al*. reported that smoking decreased retinal blood flow by 10 ± 12%^31^. Since a mild negative correlation between retinal vascular density and τ_m_ has been reported^43^, lower vascular density in the central macular in smokers^44^ may also be associated with a longer τ_m_ in the SSC. Smoking can lead to a dose-dependent increase in the hematocrit values^45^, causing the resistance of the outflow of aqueous humor in the episcleral vein, which may be related to the aforementioned elevated IOD^46^.

When the smokers group was divided into two groups, there was a slight but significant difference in age between the heavy smoker group and the other two groups. Previous report showed age-related prolongation in τ_m_ in both channels in all regions^27^. However, the differences in FLT between groups observed here were limited to specific sites, and in heavy smokers, τ_m_ showed rather a shortening in the outer macula of LSC, which would also not been explained by the age bias. Therefore, in the three-arm analysis of this study, too, it is likely that the age bias was small or negligible.

The lens autofluorescence is also known to influence the τ_m_ in FLIO, particularly in the SCC^27,47^. Clinically, no cataract was present in the study participants. Nevertheless, the possible contribution of the lens alteration in the elongation of τ_m_ in SSC cannot be completely ruled out. However, given the young age of the subjects and the fact that there was little significant difference in τ_m_ between the two smoking groups with significant age differences, the prolonged τ_m_ observed in this study is considered at least somehow related to the effect of smoking, independent of age. Further studies would be desirable in which the sub-groups are also completely age-matched.

Retinal thickness has been shown to have a positive correlation with the FLT^43^. Regarding the effect of smoking on retinal thickness, a meta-analysis by Yang et al. found no apparent association between smoking and total retinal thickness^48^. In the present study as well, no significant differences in structure and retinal thickness were detected between groups on OCT, so the possibility of changes in retinal thickness as being responsible for group differences in FLT was ruled out here.

Nicotine has been shown to increase the glycosylation of proteins in blood and tissues^49^. Advanced glycation endproducts (AGE) are known to accumulate in ocular tissues such as neurons, blood vessels, glial cells, lens, and fundus of the eye^12,50–52^. According to the report by Schweitzer *et al*., Glycated bovine serum albumin exhibited an emission maximum at 523 nm, with an average decay time of 1.7 ns and excitation at 470 nm^12^. Thus, protein glycation may be responsible for the prolonged FLT of FAFs in the fundus FLIO of diabetic patients, mainly in the SSC. Although the amount of AGEs in the retina of the subjects examined in this study cannot be directly determined, it also cannot be excluded as one of the causes of FLT prolongation in SSC in smokers. In the study by Schmidt et al. FLT in patients with non-proliferative diabetic retinopathy in SSC all components of FLT are prolonged within all ETDRS regions^53^. In the subjects in the present study, changes in the outer ring of the SSC were hardly significantly different. Also, as described above, light smokers showed rather significantly shorter τ_2_. Thus, changes in FLT in SSC in the fundus of patients with non-proliferative diabetic retinopathy and smokers do not coincide.

For LSC, differences in FLT were not as apparent as for SSC, but heavy smokers can be characterized by significantly shorter τ_m_ in the outer ring of the ETDRS grid. The main fluorophore thought to be largely responsible for autofluorescence in the LSC is lipofuscin^27^. However, accumulation of lipofuscin should rather leads to the prolongation of τ_m_ due to its long FLT^54^. Therefore, lipofuscin accumulation may be excluded as a cause of τ_m_ change in smokers in this study cohort. Indeed, studies examining the correlation between smoking and lipofuscin-related fundus autofluorescence intensity have found no apparent correlation^55^. Thus, the cause of the shortening of τ_m_ in LSC by smoking remains to be elucidated. However, the following theoretical considerations could be possible. The putative precursor of lipofuscin, a bisretinoid A2E has a quite short FLT, reportedly 0.19 ns (τ_1 =_ 0.17 ns, α_1_ = 98%, τ_2_ = 1,12 ns, α_2_ = 2%)^12^. Brogan et al. analyzed the toxic effects of nicotine and found that nicotine metabolites catalyze isomerization of 11-cis-retinal to all-trans-retinal^56^. All-trans-retinal is a precursor of A2E, thus accumulation of all-trans-retinal may lead to the biosynthesis of A2E. Thus, hypothetically, smoking may predominantly increase A2E and thus shorten the FLT in LSC. However, the study by Brogan et al. is highly experimental and mentions several steps performed in darkness. Thus, it remains unclear whether nicotine in the light-exposed retina has a considerable additional effect on retinal isomerization. Furthermore, there are currently no reports of smoking directly affecting A2E, which should be elucidated in future studies.

As discussed above, the causes of the changes in FLT cannot yet be completely specified yet. However, the results clearly show that there are characteristic differences in FLT between smokers and non-smokers. This strongly suggests the potential of FLIO to detect changes in the retinal microenvironment. Also noteworthy is that the results suggest that subtle changes caused by smoking may be clinically detectable even in healthy young adults.

Limitations of this study are the small number of subjects and the possible contribution of the biases, such as age, BMI or other systemic conditions that may affect FLT. In particular, because of the positive correlation between BMI and pack-year, future studies with larger numbers of subjects, including BMI-matched subjects, would be desirable.

Furthermore, it would be interesting to analyze, if the changes in the smoker groups are due to the years smoked (the cumulative dose) or due to the cigarettes smoked per day (acute nicotine dose) as our study does not differentiate between both. A study group with recent smokers and high daily smoking dose might therefore also be in the focus of future studies.

In summary, we found that smoking affects the FLT of the ocular fundus and this can be detected by FLIO. FLIO may provide further insights into earlier and long-term effect of smoking on the retina. There is also great potential for FLIO to detect changes in retinal status related to other lifestyle and systemic conditions, early detection of retinal diseases, and sensitive evaluation of treatment efficacy. The FLIO images of non-pathological retinas appear very similar at first glance, and it is very difficult to distinguish the differences based on the images alone. However, the numerical data therein, as shown in this study, contains highly valuable information and has the potential to detect subtle differences. Therefore, it is expected that FLIO can dramatically expand its range of applications when combined with advanced numerical data analysis techniques.

## Supplementary Information

Supplementary Information.

## Data Availability

The datasets used and analyzed during the current study are available from the corresponding author on reasonable request.
