# Supplementary material for: Impact of cigarette smoking on fluorescence lifetime of ocular fundus
Source: Sci Rep. 2023 Jul 17;13:11484. doi: 10.1038/s41598-023-37484-4 (PMC10352358; doi:10.1038/s41598-023-37484-4)
Supplement: Supplementary file 1 — Supplementary Information. [file 41598_2023_37484_MOESM1_ESM.pdf]

Impact of cigarette smoking on fluorescence lifetime of ocular fundus

Authors: Svenja Rebecca Sonntag et al.
Corresponding author: Yoko Miura

Supplementary materials

Supplementary Table S1: Mean ± standard deviation of central macular thickness of non-smokers and smokers (left table), and of non-smokers and 2 subgroups of smokers (right), with results of comparisons indicated by statistic p-values.

**Parameter**	(unit)	**Non-Smokers** (n=26)	**Smokers** (n=28)	**p**
MT C OD	(µm)	280 ± 13.3	285 ± 20.7	0.29
MT C OS	(µm)	281 ± 16.4	283 ± 22.5	0.70
MT N1 OD	(µm)	351 ± 12.1	356 ± 15.5	0.19
MT N1 OS	(µm)	351 ± 13.2	356 ± 16.6	0.23
MT N2 OD	(µm)	319 ± 14.5	313 ± 56.6	0.60
MT N2 OS	(µm)	321 ± 12.9	326 ± 14.1	0.16
MT S1 OD	(µm)	349 ± 12.9	353 ± 15.9	0.31
MT S1 OS	(µm)	350 ± 12.7	355 ± 16.7	0.23
MT S2 OD	(µm)	312 ± 13.8	319 ± 15.7	0.07
MT S2 OS	(µm)	310 ± 13.9	317 ± 14.0	0.07
MT T1 OD	(µm)	337 ± 12.4	341 ± 16.2	0.37
MT T1 OS	(µm)	336 ± 11.8	341 ± 16.7	0.21
MT T2 OD	(µm)	289 ± 12.4	295 ± 13.3	0.07
MT T2 OS	(µm)	288 ± 13.4	293 ± 13.5	0.12
MT I1 OD	(µm)	347 ± 12.5	350 ± 14.6	0.41
MT I1 OS	(µm)	347 ± 12.6	351 ± 15.7	0.33
MT I2 OD	(µm)	297 ± 12.0	300 ± 13.8	0.42
MT I2 OS	(µm)	298 ± 12.4	303 ± 14.0	0.14
MT IR OD	(µm)	1384 ± 47.5	1400 ± 60.3	0.29
MT IR OS	(µm)	1385 ± 48.5	1403 ± 64.3	0.23
MT OR OD	(µm)	1217 ± 49.4	1228 ± 79.5	0.55
MT OR OS	(µm)	1217 ± 48.7	1240 ± 51.8	0.11

**Parameter**	(unit)	**Group 0 (G0)** Non-smokers (n=26)	**Group 1 (G1)** Smokers PY <7.11 (n=14)	**Group 2 (G2)** Smokers PY ≥7.11 (n=14)	**p**
MT C OD	(µm)	280 ± 13.3	286 ± 23.9	284 ± 17.7	0.53
MT C OS	(µm)	281 ± 16.4	285 ± 24.2	281 ± 21.4	0.81
MT N1 OD	(µm)	351 ± 12.1	357 ± 15.7	356 ±15.9	0.42
MT N1 OS	(µm)	351 ± 13.2	356 ± 15.0	356 ± 18.7	0.49
MT N2 OD	(µm)	319 ± 14.5	302 ± 78.4	324 ± 15.4	0.51
MT N2 OS	(µm)	321 ± 12.9	325 ± 12.2	328 ± 16.1	0.33
MT S1 OD	(µm)	349 ± 12.9	354 ± 15.5	353 ± 16.8	0.59
MT S1 OS	(µm)	350 ± 12.7	356 ± 13.9	354 ± 19.7	0.50
MT S2 OD	(µm)	312 ± 13.8	317 ± 13.9	322 ± 17.5	0.13
MT S2 OS	(µm)	310 ± 13.9	315 ± 11.5	320 ± 16.0	0.12
MT T1 OD	(µm)	337 ± 12.4	342 ± 15.7	339 ± 17.2	0.58
MT T1 OS	(µm)	336 ± 11.8	341 ± 15.3	341 ± 18.5	0.44
MT T2 OD	(µm)	289 ± 12.4	292 ± 11.2	299 ± 14.8	0.09
MT T2 OS	(µm)	288 ± 13.4	291 ± 11.0	295 ± 15.8	0.34
MT I1 OD	(µm)	347 ± 12.5	350 ± 11.8	349 ± 17.5	0.70
MT I1 OS	(µm)	347 ± 12.6	350 ± 11.5	352 ± 19.4	0.57
MT I2 OD	(µm)	297 ± 12.0	298 ± 10.8	302 ± 16.5	0.51
MT I2 OS	(µm)	298 ± 12.4	301 ± 9.8	306 ± 17.2	0.21
MT IR OD	(µm)	1384 ± 47.5	1404 ± 57.0	1397 ± 65.4	0.55
MT IR OS	(µm)	1385 ± 48.5	1404 ± 54.6	1403 ± 74.8	0.44
MT OR OD	(µm)	1217 ± 49.4	1209 ± 91.7	1249 ± 60.7	0.25
MT OR OS	(µm)	1217 ± 48.7	1231 ± 40.9	1248 ± 61.2	0.19

MT: macular thickness, C: center, N: nasal, S: superior, T: temporal, I: inferior, 1: inner area, 2: outer area, IR: inner ring, OR: outer ring, OD: oculus dexter (right eye), OS: oculus sinister (left eye)

Supplementary Table S2: Correlation between OD and OS regarding all FLT components in all areas in both channels

OD vs OS

channel	parameter	area	OD mean (SD)	OS Mean (SD)	Pearson r	P-value
SSC	τ_m	C	133.6 (19.44)	132.9 (19.70)	0.887	<0.0001
		IR	215 (17.10)	214.8 (20.24)	0.862	<0.0001
		OR	241.8 (14.07)	241.1 (16.73)	0.812	<0.0001
	τ_1	C	96.58 (12.18)	95.94 (11.42)	0.909	<0.0001
		IR	120.9 (19.98)	120.3 (20.34)	0.972	<0.0001
		OR	134 (27.49)	133.3 (28.12)	0.988	<0.0001
	τ_2	C	980 (659.1)	974.9 (659.6)	0.992	<0.0001
		IR	1052 (708)	1049 (709)	0.997	<0.0001
		OR	1033 (695.1)	1029 (694.8)	0.997	<0.0001
LSC	τ_m	C	201 (24.02)	199.1 (24.07)	0.861	<0.0001
		IR	221.2 (11.80)	218.9 (13.69)	0.814	<0.0001
		OR	238.5 (13.74)	236.2 (15.69)	0.826	<0.0001
	τ_1	C	124.1 (21.32)	123 (21.26)	0.988	<0.0001
		IR	139.8 (31.58)	138.4 (31.75)	0.99	<0.0001
		OR	147 (36.78)	145.7 (37.00)	0.992	<0.0001
	τ_2	C	719.5 (460.3)	716.2 (460.0)	0.992	<0.0001
		IR	784.6 (507.4)	779.8 (505.7)	0.998	<0.0001
		OR	778.6 (502.5)	773.7 (500.6)	0.999	<0.0001

Supplementary Figure 1: Bland-Altman plot (percentage difference against average) to compare the τ_m in right and left eyes

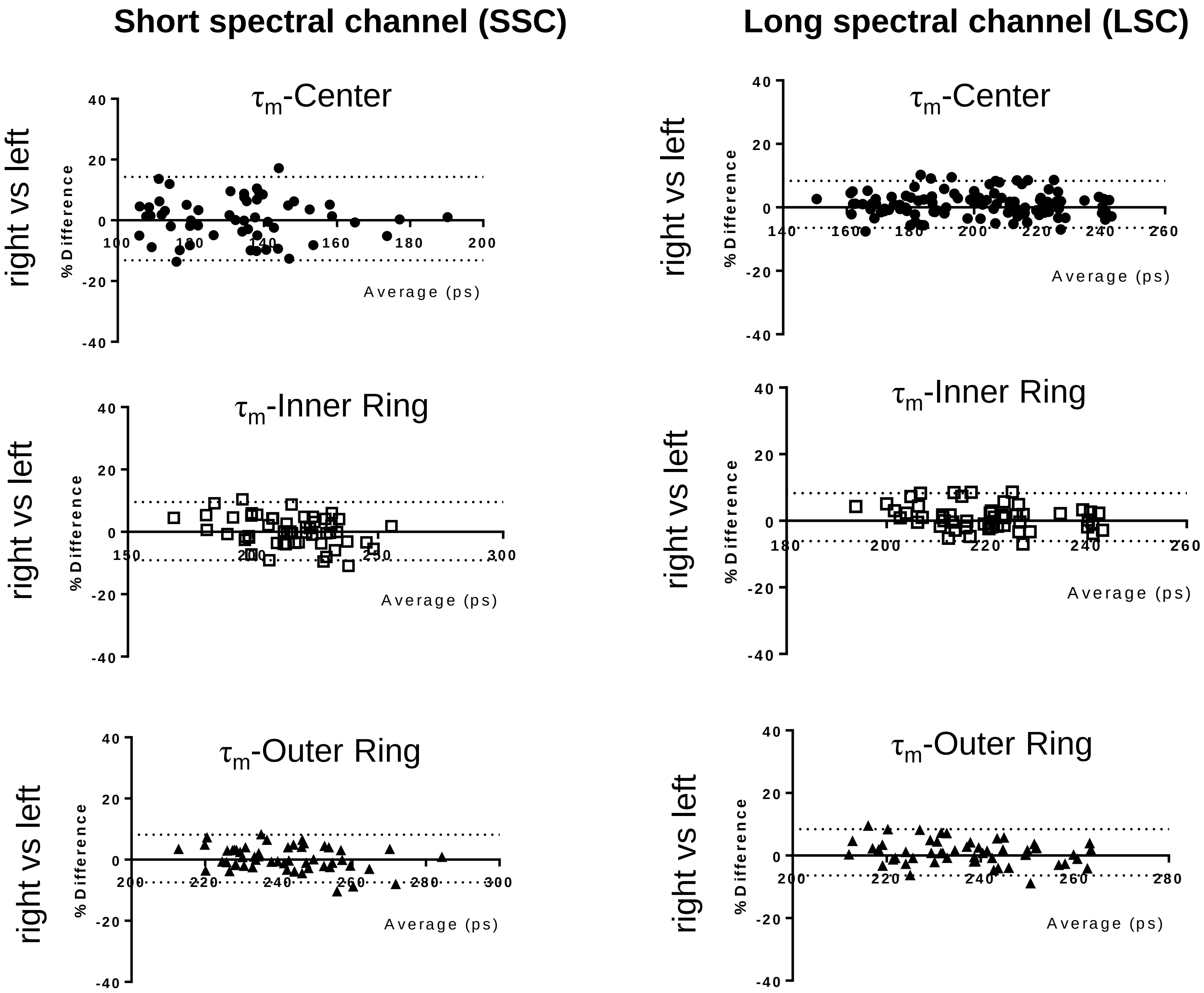

Supplementary Table S3: Mean ± standard deviation of τ_1 (in ps) in SSC (top) and in LSC (bottom) for each area of ETDRS grid in the right eye of the groups of non-smokers and smokers, p-values from post hoc test after repeated measure ANOVA, and the results of correlation analysis (r: correlation coefficient) between the τ_1 in SSC and the number of pack-year, years-smoked and retinal thickness at the corresponding area.
* p < 0.05, ** p < 0.01, *** p < 0.001.

	**area**	**τ_1 (ps)**		**post hoc p**	**τ_1 (ps)**			**post hoc p**			**correlation coefficient (r)**			**area**
		non-smokers (n=26)	smokers (n=28)		Group 0 (G0) Non-smokers (n=26)	Group 1(G1) Smokers PY <7.11 (n=14)	Group 2 (G2) Smokers PY ≥7.11 (n=14)	G0 vs G1	G0 vs G2	G1 vs G2	pack-year	years-smoked	retinal thickness	
**SSC**	C	93.6 ± 11	99.4 ± 12.7	0.080	93.6 ± 11	97.1 ± 10.4	102 ± 14.7	0.374	0.047*	0.324	0.399**	0.350**	0.011	C
	N1	128 ± 11.1	135 ± 11.5	0.039*	128 ± 11.1	133 ± 10.7	136 ± 12.5	0.198	0.035*	0.456	0.363**	0.420**	-0.072	N1
	N2	152 ± 7.1	155 ± 8.0	0.220	152 ± 7.1	154 ± 8.3	155 ± 7.9	0.432	0.224	0.702	0.276*	0.398**	0.113	N2
	S1	133 ± 10.2	139 ± 9.6	0.040*	133 ± 10.2	138 ± 8.4	140 ± 10.7	0.172	0.046*	0.565	0.357**	0.408**	-0.177	S1
	S2	153 ± 5.1	154 ± 6.1	0.474	153 ± 5.1	154 ± 6.0	154 ± 6.3	0.643	0.483	0.833	0.218	0.335*	-0.003	S2
	T1	128 ± 10.8	134 ± 10.6	0.033*	128 ± 10.8	132 ± 10.1	136 ± 11.2	0.224	0.022*	0.329	0.401**	0.449***	-0.113	T1
	T2	147 ± 6.6	151 ± 7.0	0.058	147 ± 6.6	150 ± 7.6	151 ± 6.6	0.190	0.072	0.658	0.351**	0.445***	-0.032	T2
	I1	129 ± 11.3	136 ± 11.0	0.029*	129 ± 11.3	133 ± 9.9	138 ± 11.9	0.222	0.017*	0.288	0.410**	0.461***	-0.131	I1
	I2	153 ± 7.3	157 ± 8.2	0.076	153 ± 7.3	156 ± 9.6	158 ± 6.7	0.234	0.087	0.636	0.335*	0.446***	0.017	I2
	Inner Ring	130 ± 10.7	136 ± 10.5	0.033*	130 ± 10.7	134 ± 9.6	138 ± 11.5	0.197	0.026*	0.391	0.388**	0.441***	-0.123	Inner Ring
	Outer Ring	151 ± 6.2	154 ± 7.1	0.135	151 ± 6.2	154 ± 7.7	155 ± 6.8	0.319	0.149	0.688	0.310*	0.425**	0.071	Outer Ring

	**area**	**τ_1 (ps)**		**post hoc p**	**τ_1 (ps)**			**post hoc p**			**correlation coefficient (r)**			**area**
		non-smokers (n=26)	smokers (n=28)		Group 0 (G0) Non-smokers (n=26)	Group 1(G1) Smokers PY <7.11 (n=14)	Group 2 (G2) Smokers PY ≥7.11 (n=14)	G0 vs G1	G0 vs G2	G1 vs G2	pack-year	years-smoked	retinal thickness	
**LSC**	C	136 ± 8.3	140 ± 9.2	0.096	136 ± 8.3	140 ± 10.4	140 ± 8.2	0.184	0.164	0.954	0.287*	0.300*	0.063	C
	N1	161 ± 6.5	162 ± 8.1	0.767	161 ± 6.5	163 ± 9.4	161 ± 6.9	0.529	0.884	0.497	0.103	0.177	0.037	N1
	N2	177 ± 6.5	174 ± 9.2	0.206	177 ± 6.5	176 ± 10.0	173 ± 8.5	0.593	0.125	0.373	-0.069	0.011	-0.023	N2
	S1	163 ± 6.2	163 ± 7.2	0.961	163 ± 6.2	164 ± 8.3	162 ± 6.1	0.743	0.683	0.518	0.073	0.110	-0.004	S1
	S2	174 ± 6.2	171 ± 7.4	0.104	174 ± 6.2	172 ± 8.3	169 ± 6.3	0.498	0.045*	0.235	-0.118	-0.093	-0.005	S2
	T1	160 ± 6.4	161 ± 7.7	0.949	160 ± 6.4	162 ± 9.2	159 ± 5.8	0.552	0.624	0.343	0.062	0.106	-0.066	T1
	T2	170 ± 6.5	168 ± 8.3	0.233	170 ± 6.5	170 ± 9.8	165 ± 5.8	0.996	0.049*	0.084	-0.120	-0.095	-0.023	T2
	I1	162 ± 6.0	162 ± 8.1	0.697	162 ± 6.0	163 ± 9.7	161 ± 6.2	0.459	0.917	0.459	0.114	0.189	-0.017	I1
	I2	175 ± 6.3	174 ± 9.3	0.628	175 ± 6.3	176 ± 11.3	172 ± 6.5	0.765	0.274	0.223	0.001	0.094	0.107	I2
	Inner Ring	162 ± 6.1	162 ± 7.7	0.855	162 ± 6.1	163 ± 9.1	161 ± 6.2	0.557	0.773	0.443	0.090	0.149	-0.010	Inner Ring
	Outer Ring	174 ± 6.1	172 ± 8.3	0.243	174 ± 6.1	174 ± 9.7	170 ± 6.4	0.821	0.091	0.195	-0.077	-0.018	-0.001	Outer Ring

Supplementary Table S4: Mean ± standard deviation of $\boldsymbol{\tau_2}$ (in ps) in SSC (top) and in LSC (bottom) for each area of ETDRS grid in the right eye of the groups of non-smokers and smokers, p-values from post Hoc test after repeated measure ANOVA, and the results of correlation analysis (r: correlation coefficient) between the τ_1 in SSC and the number of pack-year, years-smoked and retinal thickness at the corresponding area.
* p < 0.05, ** p < 0.01, *** p < 0.001.

	**area**	τ_2 (ps)		**post hoc p**	τ_2 (ps)			**post hoc p**			**correlation coefficient (r)**			**area**
		non-smokers (n=26)	smokers (n=28)		Group 0 (G0) Non-smokers (n=26)	Group 1(G1) Smokers PY <7.11 (n=14)	Group 2 (G2) Smokers PY ≥7.11 (n=14)	G0 vs G1	G0 vs G2	G1 vs G2	pack-year	years-smoked	retinal thickness	
**SSC**	C	1476 ± 142	1432 ± 134	0.245	1476 ± 142	1371 ± 101	1493 ± 137	0.019*	0.698	0.017*	0.045	- 0.064	-0.024	C
	N1	1578 ± 100	1551 ± 105	0.335	1578 ± 100	1507 ± 84.4	1595 ± 108	0.034*	0.608	0.022*	0.022	- 0.076	0.195	N1
	N2	1575 ± 111	1550 ± 111	0.396	1575 ± 111	1506 ± 89.9	1593 ± 116	0.057	0.626	0.038*	0.002	- 0.077	0.061	N2
	S1	1606 ± 101	1578 ± 104	0.326	1606 ± 101	1533 ± 79.4	1623 ± 108	0.029*	0.590	0.018*	0.045	- 0.082	0.210	S1
	S2	1550 ± 121	1521 ± 118	0.375	1550 ± 121	1480 ± 98.0	1563 ± 124	0.074	0.753	0.066	- 0.013	- 0.115	0.221	S2
	T1	1555 ± 105	1528 ± 106	0.361	1555 ± 105	1485 ± 84.2	1572 ± 111	0.042*	0.614	0.027*	0.030	- 0.079	0.111	T1
	T2	1518 ± 117	1501 ± 109	0.568	1518 ± 117	1465 ± 98.9	1536 ± 110	0.154	0.628	0.096	0.000	- 0.094	0.345*	T2
	I1	1574 ± 107	1550 ± 109	0.413	1574 ± 107	1504 ± 91.3	1596 ± 109	0.046*	0.531	0.023*	0.044	- 0.054	0.283*	I1
	I2	1546 ± 121	1525 ± 116	0.529	1546 ± 121	1483 ± 101	1568 ± 118	0.105	0.564	0.056	0.021	- 0.070	0.364**	I2
	Inner Ring	1578 ± 103	1552 ± 105	0.356	1578 ± 103	1507 ± 84.1	1596 ± 108	0.036*	0.582	0.022*	0.035	- 0.073	0.202	Inner Ring
	Outer Ring	1547 ± 116	1524 ± 112	0.457	1547 ± 116	1484 ± 95.6	1565 ± 115	0.088	0.637	0.058	0.002	- 0.090	0.200	Outer Ring

	**area**	τ_2(ps)		**post hoc p**	τ_2 (ps)			**post hoc p**			**correlation coefficient (r)**			**area**
		non-smokers (n=26)	smokers (n=28)		Group 0 (G0) Non-smokers (n=26)	Group 1(G1) Smokers PY <7.11 (n=14)	Group 2 (G2) Smokers PY ≥7.11 (n=14)	G0 vs G1	G0 vs G2	G1 vs G2	pack-year	years-smoked	retinal thickness	
**LSC**	C	1061 ± 73.0	1045 ± 49.3	0.350	1061 ± 73.0	1030 ± 47.8	1060 ± 47.7	0.133	0.974	0.196	0.015	-0.088	0.103	C
	N1	1163 ± 55.5	1146 ± 51.8	0.265	1163 ± 55.5	1141 ± 49.9	1152 ± 54.9	0.224	0.544	0.590	-0.113	-0.167	0.147	N1
	N2	1177 ± 44.5	1154 ± 47.8	0.076	1177 ± 44.5	1153 ± 47.5	1154 ± 49.9	0.137	0.158	0.944	-0.149	-0.152	0.124	N2
	S1	1173 ± 61.1	1155 ± 51.8	0.249	1173 ± 61.1	1147 ± 47.6	1163 ± 56.4	0.179	0.590	0.475	-0.090	-0.179	0.146	S1
	S2	1144 ± 49.0	1122 ± 45.1	0.104	1144 ± 49.0	1122 ± 41.9	1123 ± 49.6	0.165	0.205	0.913	-0.127	-0.179	0.218	S2
	T1	1145 ± 51.9	1130 ± 48.0	0.286	1145 ± 51.9	1127 ± 44.9	1134 ± 52.5	0.281	0.509	0.711	-0.103	-0.168	0.044	T1
	T2	1131 ± 44.4	1116 ± 42.1	0.188	1131 ± 44.4	1120 ± 41.9	1112 ± 43.4	0.418	0.181	0.638	-0.150	-0.183	0.227	T2
	I1	1164 ± 53.8	1149 ± 51.4	0.290	1164 ± 53.8	1143 ± 49.6	1155 ± 54.4	0.232	0.595	0.556	-0.096	-0.147	0.167	I1
	I2	1162 ± 45.2	1147 ± 45.3	0.231	1162 ± 45.2	1148 ± 46.6	1145 ± 45.7	0.373	0.288	0.878	-0.096	-0.093	0.307*	I2
	Inner Ring	1161 ± 55.0	1145 ± 50.2	0.267	1161 ± 55.0	1140 ± 47.5	1151 ± 54.1	0.221	0.556	0.573	-0.101	-0.167	0.121	Inner Ring
	Outer Ring	1153 ± 44.6	1135 ± 43.8	0.128	1153 ± 44.6	1136 ± 43.6	1134 ± 45.7	0.236	0.193	0.915	-0.134	-0.156	0.201	Outer Ring
